# Effects of Prior Antiplatelet Therapy on Mortality, Functional Outcome, and Hematoma Expansion in Intracerebral Hemorrhage: An Updated Systematic Review and Meta-Analysis of Cohort Studies

**DOI:** 10.3389/fneur.2021.691357

**Published:** 2021-08-23

**Authors:** Yujie Wu, Donghang Zhang, Hongyang Chen, Bin Liu, Cheng Zhou

**Affiliations:** ^1^Laboratory of Anesthesia and Critical Care Medicine, Translational Neuroscience Center, National Clinical Research Center for Geriatrics, West China Hospital of Sichuan University, Chengdu, China; ^2^Department of Anesthesiology, West China Hospital of Sichuan University, Chengdu, China

**Keywords:** antiplatelet therapy, intracerebral hemorrhage, mortality, functional outcome, hematoma expansion

## Abstract

**Background and Objective:** Antiplatelet therapy (APT) is widely used and believed to be associated with increased poor prognosis by promoting bleeding in patients with intracerebral hemorrhage (ICH). We performed a systematic review and meta-analysis to determine whether prior APT is associated with mortality, functional outcome, and hematoma expansion in ICH patients.

**Methods:** The PubMed, Embase, and Web of Science databases were searched for relevant published studies up to December 11, 2020. Univariate and multivariable adjusted odds ratios (ORs) were pooled using a random effects model. Cochran's chi-squared test (Cochran's Q), the *I*^2^ statistic, and meta-regression analysis were used to evaluate the heterogeneity. Meta-regression models were developed to explore sources of heterogeneity. Funnel plots were used to detect publication bias. A trim-and-fill method was performed to identify possible asymmetry and assess the robustness of the conclusions.

**Results:** Thirty-one studies fulfilled the inclusion criteria and exhibited a moderate risk of bias. Prior APT users with intracerebral hemorrhage (ICH) had a slightly increased mortality in both univariate analyses [odds ratio (OR) 1.39, 95% CI 1.24–1.56] and multivariable adjusted analyses (OR 1.41, 95% CI 1.21–1.64). The meta-regression indicated that for each additional day of assessment time, the adjusted OR for the mortality of APT patients decreased by 0.0089 (95% CI: −0.0164 to −0.0015; *P* = 0.0192) compared to that of non-APT patients. However, prior APT had no effects on poor function outcome (pooled univariate OR: 0.99, 95% CI 0.59–1.66; pooled multivariable adjusted OR: 0.93, 95% CI 0.87–1.07) or hematoma growth (pooled univariate OR: 1.23, 95% CI 0.40–3.74, pooled multivariable adjusted OR: 0.94, 95% CI 0.24–3.60).

**Conclusions:** Prior APT was not associated with hematoma expansion or functional outcomes, but there was modestly increased mortality in prior APT patients. Higher mortality of prior APT patients was related to the strong influence of prior APT use on early mortality.

**Systematic Review Registration:**PROSPERO Identifier [CRD42020215243].

## Introduction

Spontaneous intracerebral hemorrhage (ICH) in patients taking antiplatelet therapy (APT) is common in routine clinical practice (1, 2). Antiplatelet therapy (APT) has attracted wide attention because of its beneficial effects on cardiovascular and cerebrovascular diseases (3). Approximately 20% to 30% of patients with ICH are on APT (4, 5). However, prior APT is believed to be associated with increased mortality and poor prognosis due to the promotion of bleeding in patients with ICH (6). Several studies have reported that ongoing hemorrhage expansion is an independent predictor of increased mortality and poor functional outcome following ICH (7).

Previous studies regarding the prognosis of prior APT in patients with ICH have shown conflicting results. Some suggest that an increased risk of death and poor outcome are associated with prior APT (5, 8, 9), while others suggest the opposite association (10–13). A meta-analysis published in 2010 (4) found higher mortality in ICH patients with prior APT. Recently, several large cohort studies reported that prior APT was not associated with significant death and disability (10, 12, 13). Overall, whether prior APT is associated with higher mortality, poor outcome, or hematoma expansion in ICH patients remains unclear. Given the conflicting data between APT and ICH outcomes, the current American Heart Association and European Stroke Organization guidelines for the routine use of platelet transfusion after ICH are inconclusive (14). Meanwhile, it is not clear whether APT use is related to hematoma enlargement. Thus, it is worthwhile to perform an updated systematic review and meta-analysis to determine the correlation between prior APT use and ICH outcomes.

## Methods

### Search Strategy

This meta-analysis was registered in the International Prospective Register of Systematic Reviews (PROSPERO; Registration NO. CRD42020215243) and conducted following the guidelines of the Cochrane Handbook for Systematic Reviews of Intervention and the PRISMA statements (15). Two authors systematically searched the following databases from inception to December 11, 2020: PubMed, Embase, and Web of Science. The following terms were used to identify eligible studies: (“ICH” OR “intracerebral hemorrhage” OR “intracerebral”) AND (“APT” OR “antiplatelet”). No language restriction was applied. In addition, we also performed a manual search of the references in relevant articles to retrieve eligible studies.

### Inclusion Criteria

The inclusion criteria were as follows: (1) cohort studies included consecutive patients with the primary outcome or the secondary outcome of ICH; (2) ICH patients were all verified by computed tomography or magnetic resonance imaging; (3) prior APT was one of the influencing analysis factors; (4) the adjusted or unadjusted odds ratio (OR) with aspect among mortality, poor function outcome, and hematoma growth between ICH patients with and without prior APT could be acquired directly or by calculation; and (5) primary outcome: mortality after intracranial hemorrhage in consecutive patients; secondary outcomes: (a) poor function outcome, defined as being within a specific scoring range using widely accepted validated scales [the modified Rankin Scale (mRS) or the Glasgow Scale Score (GSS)]; (b) hematoma growth was defined as an increase in the baseline hematoma volume by either 33% or >6 ml on the interval CT scan performed within 72 h.

### Exclusion Criteria

(1) Patients included secondary cerebral hemorrhage caused by trauma, tumor, aneurysm rupture, or arteriovenous malformation. (2) Studies cannot strictly separate ICH patients with APT and without APT due to a lack of detailed information. (3) Studies without enough information to judge the effectiveness of the statistical methods.

### Study Selection

Two authors (YW and DZ) independently reviewed the identified studies. Full texts of potentially relevant articles were retrieved after screening titles and abstracts. Any disagreement was discussed with the third author (HC).

### Data Extraction

Two authors (YW and DZ) independently extracted the following data from eligible studies: study characteristics (first author, year of publication), study range (single-center or multicenter), study type (prospective or retrospective), study continent, study reputation (the mean-centered impact score of the journal the study was published in), patient age, and assessment time. Since the combination of APT and other anticoagulant drugs would lead to a change in the drug mechanism, we extracted the data of the patients who take APT alone in this paper. Meanwhile, we extracted data on mortality, poor function outcome, and hematoma growth at all time points in all included studies. Mortality data were divided into early time, 30-, 90-day, and discharge groups to compare the differences between groups.

### Risk of Bias Assessment

Risk of bias was assessed by two investigators (YW and DZ) with the Robins-I tool for non-randomized studies. The following domains for the non-randomized studies were evaluated: confounding, selection of participants, departure from intended interventions, missing data, measurement of outcomes, and selective reporting at low, moderate, serious, or critical risk. These domains were combined to result in an overall risk of bias judgment as low, moderate, serious, or critical. Discrepancies in risk of bias assessment were resolved *via* discussion (16).

### Statistical Analysis

This meta-analysis was performed using the R software (version 4.0.2, 64 bits, The Cochrane Collaboration, Oxford, UK). Raw data containing valid results were calculated as odds ratios for statistical analysis. To ensure the reliability of the study, we separately pooled the adjusted OR or unadjusted OR with a 95% confidence interval (95% CI) as the effect size of this meta-analysis. Cochran's chi-squared test (Cochran's Q) and *I*^2^-test were used to analyze heterogeneity among the studies. According to the Cochrane Review guidelines, the threshold for heterogeneity is an *I*^2^ < 50% and a *P* < 0.1 and indicated using a random effects model in OR computation rather than a fixed effects model (17). Furthermore, based on a literature review and clinical experience, the possible variables that may cause heterogeneity, including publication year, study center, study type, study continent, and study reputation, were analyzed by univariate meta-regression. *P* < 0.05 was considered the cause of heterogeneity. On the other hand, we performed subgroup analysis based on the assessment time. In addition, we separately performed a meta-regression analysis to explore the relationship between assessment time and mortality under prior APT use. Sensitivity analysis was then carried out by excluding each study one by one. Publication bias was assessed both visually evaluating the symmetry of the funnel plot and mathematically using the Egger regression intercept for outcomes. *P*-values <0.05 were identified as significant publication bias (18).

## Results

### Results of the Literature Search

**Figure 1:**
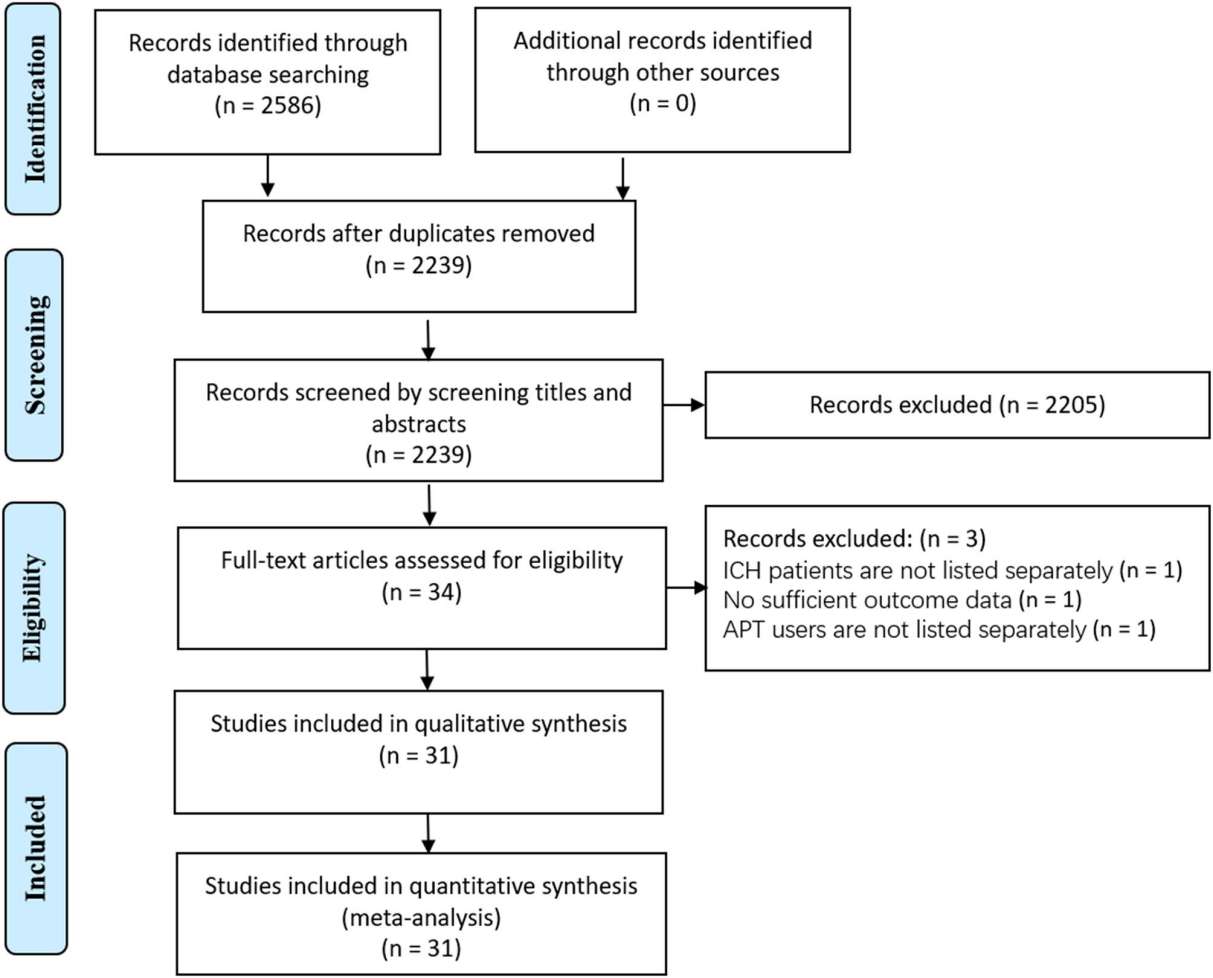
Study flowchart.

The search of electronic bibliographic sources retrieved a total of 2,586 studies. After screening the title, abstract, and full text, 31 cohort studies met our eligibility criteria and were included in the analysis. The PRISMA flow diagram for the selection is presented in Figure 1.

### Study Characteristics

All 31 studies (5, 6, 8–13, 19–41) had an observational design, of which 17 studies (6, 8–10, 12, 13, 20, 25, 26, 28–30, 32, 35, 38, 40, 41) were retrospective relying on medical records, and the other 14 studies (5, 11, 19, 21–24, 27, 31, 33, 34, 36, 37, 39) were prospective cohort studies. Twenty studies (6, 10, 11, 13, 20, 22, 23, 25–31, 33, 35, 36, 38, 39, 41) were single-center studies, while the rest were conducted in more than one institution. Among the studies, 14 were conducted in Europe (5, 10, 11, 13, 21, 23–25, 27–29, 35, 39, 40), 6 were conducted in America (12, 22, 30, 31, 33, 39), and 11 were conducted in Asia (6, 9, 19, 20, 26, 32, 34, 36–38, 41). In total, 219,726 patients were included, of which 50,285 underwent APT therapy (weighted mean proportion 22.9%, range 4.3–44.9%). Generally, patients on APT were older and had more cerebrovascular risk factors across most studies (5, 9–13, 24–36, 38, 39). The characteristics of the included studies are summarized in Table 1.

**Table 1:** Characteristics of eligible studies.

**References**	**Study type**	**Continent**	**Study population**	**No**.	**Mean age (SD)**	**Apt mean age (SD)**	**Not apt mean age (SD)**	**Male %**	**Pre-ICH APT, %**	**Time of assessment**	**All mortality (%)**	**Definition of poor outcome**
Camps-Renom et al. (11)	Prospectively	Europe	Single center	223	72.5 (13)	77.3 (10)	70.1 (3.7)	54.3	74 (33.2)	90 days	31.4	mRS 3-6
Gulati et al. (40)	Retrospectively	Europe	Multicenter	19,921	NR	66.1	NR	NR	5,865 (29.4)	90 days	25.1	mRS 3-6
Hallevy et al. (20)	Retrospectively	Asia	Single center	169	71.2	NR	NR	54.4	18.5	Discharge	33	mRS 4–6
Nilsson et al. (21)	Prospectively	Europe	Multicenter	338	74	NR	NR	56	74 (21.9)	30 days	36	NR
Rosand et al. (22)	Prospectively	America	Single center	435	74.4 (9.3)	NR	NR	48.2	139 (32.0)	90 days	32	NR
Toyoda et al. (6)	Retrospectively	Asia	Single center	251	66	NR	NR	60.6	57(42.1)	Discharge	12.4	NR
Roquer et al. (23)	Prospectively	Europe	Single center	387	71.6 (12.5)	NR	NR	55.2	47 (24.2)	30 days	26.3	NR
Foerch et al. (24)	Prospectively	Europe	Multicenter	1,483	72 (12)	75 (10)	70 (14)	52	441 (26)	Discharge	22.7	mRS 3-6
Karlikaya et al. (25)	Retrospectively	Europe	Single center	664	NR	67.1 (12.5)	65.8 (13.3)	NR	40 (6.0)	21 days	28	mRS 3-6
Saloheimo et al. (26)	Retrospectively	Asia	Single center	182	NR	71.6 (11.2)	65.6 (11.1)	49.5	44 (24.1)	90 days	32.7	NR
Caso et al. (27)	Prospectively	Europe	Single center	457	NR	78.9 (9.0)	73.8 (9.4)	58	94 (20.5)	Discharge	23.2	GOS 1–3
Lacut et al. (28)	Retrospectively	Europe	Single center	138	NR	70.5	61.5	60.9	30 (21.7)	7/30/90 days	20.29	mRS 4–6
Hanger et al. (29)	Retrospectively	Europe	Single center	223	NR	75.7	69.9	48.9	91(39.2)	8/14/28days	42.3	mRS 3-6
Creutzfeldt et al. (30)	Retrospectively	America	Single center	368	72.5	70 (12)	62 (17)	50.3	121 (31.3)	Discharge	34.5	mRS 3-6
Sansing et al. (31)	Prospectively	America	Single center	282	NR	71	63	66	70 (24.8)	90 days	17.2	mRS 3-6
Toyoda et al. (32)	Retrospectively	Asia	Multicenter	918	NR	71 (10)	65 (13)	59.3	180 (19.6)	21 days	13.2	mRS 3-6
Stead et al. (33)	Prospectively	America	Single center	178	NR	79	66	49.4	80 (44.9)	7/30 days	27	mRS 2-6
Balci et al. (34)	Prospectively	Asia	Multicenter	337	NR	70.1 (10.9)	67.2 (11.2)	44.5	48 (14.2)	Discharge	36.5	mRS 3-6
Kuramatsu et al. (35)	Retrospectively	Europe	single-center	210	69.6 (11.7)	72.2 (11.0)	67.9 (11.9)	51.9	83 (39.5)	90 days	31.4	mRS 4–6
Yildiz et al. (36)	Prospectively	Asia	Single center	153	66 (12)	70 (11)	64 (12)	61.4	52 (34)	NR	NR	mRS 3-6
Chen et al. (10)	Retrospectively	Europe	Single center	1,927	NR	68.4 (11.7)	61.5 (14.5)	63.7	232 (12)	30 days	15	NR
Mansouri et al. (37)	Prospectively	Asia	Multicenter	90	64.6	NR	NR	NR	26 (28.9)	90 days	47	NR
Yang et al. (38)	Retrospectively	Asia	Single center	333	NR	65.4(12.6)	57.5(13.9)	40	68(20.4)	Discharge	46.7	GCS worsen
Stein et al. (5)	Prospectively	Europe	Multicenter	7,051	NR	77.2 (10.0)	70.1 (14.1)	48	2,113 (30.0)	Discharge	23.2	NR
Roquer et al. (39)	Prospectively	Europe	Single center	440	NR	80	74	50.9	147(33.4)	1/90 days	NR	NR
Khan et al. (8)	Retrospectively	America	Multicenter	82,576	64	NR	NR	NR	28,277 (34.2)	Discharge	24.2	mRS 3-6
Hokari et al. (41)	Retrospectively	Asia	Single center	429	NR	NR	NR	58.3	64 (14.9)	30 days	NR	NR
van Ginneken et al. (13)	Retrospectively	Europe	Single center	343	NR	77	72	49.3	99 (29)	Discharge	10	mRS 5-6
Liu et al. (9)	Retrospectively	Asia	Multicenter	97,355	NR	69	NR	64.4	11,351 (11.7)	Discharge	NR	NR
Murthy et al. (12)	Retrospectively	America	Multicenter	1,420	NR	66.5 (11.6)	61.3 (12.5)	NR	284 (20)	Discharge	NR	mRS 4–6
Wong et al. (19)	Prospectively	Asia	Multicenter	783	61.3 (15.02)	NR	NR	69	34 (4.3)	Discharge	29.8	mRS 4–6

*NR, not reported; APT, antiplatelet therapy; ICH, intracerebral hemorrhage; mRS, modified Rankin Scale; GSS, Glasgow Scale Score*.

### Risk of Bias in Included Studies

**Figure 2:**
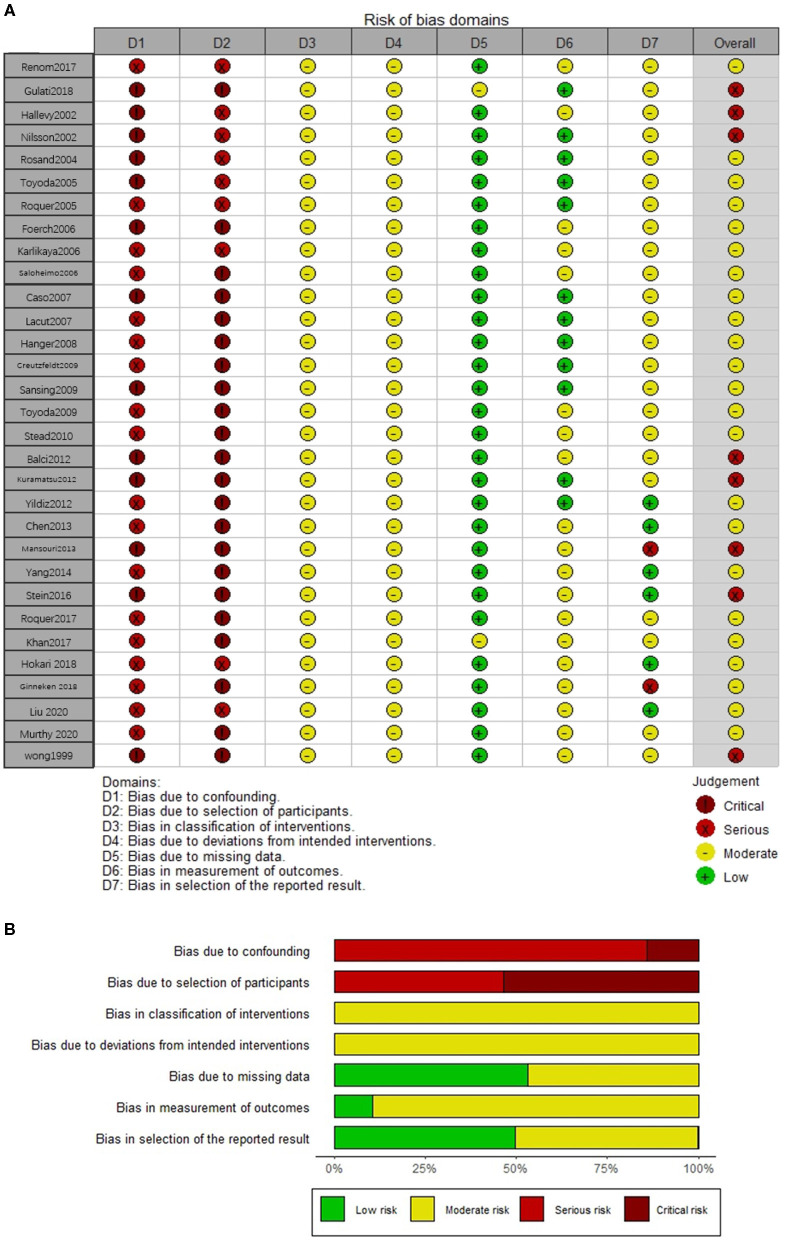
The risk of bias assessment of each included study **(A)** and weighted summary of the risk of bias **(B)**.

All included studies were rated as having a moderate or serious risk of bias with the ROBINS-I tool (Figures 2A,B). Twenty-three studies (6, 8–13, 22–33, 36, 38, 39, 41) had a moderate risk of bias since they were generally involved with adjustment for confounding, although possible residual confounding could not be excluded. The other seven studies (5, 19–21, 34, 35, 40) were judged as having a serious risk of bias, mainly due to a lack of control for confounding and measurement bias in studies relying on retrospective medical records.

### Results of Meta-Analysis

#### Primary Outcome

##### Effects of Prior APT on Mortality of ICH Patients

**Figure 3:**
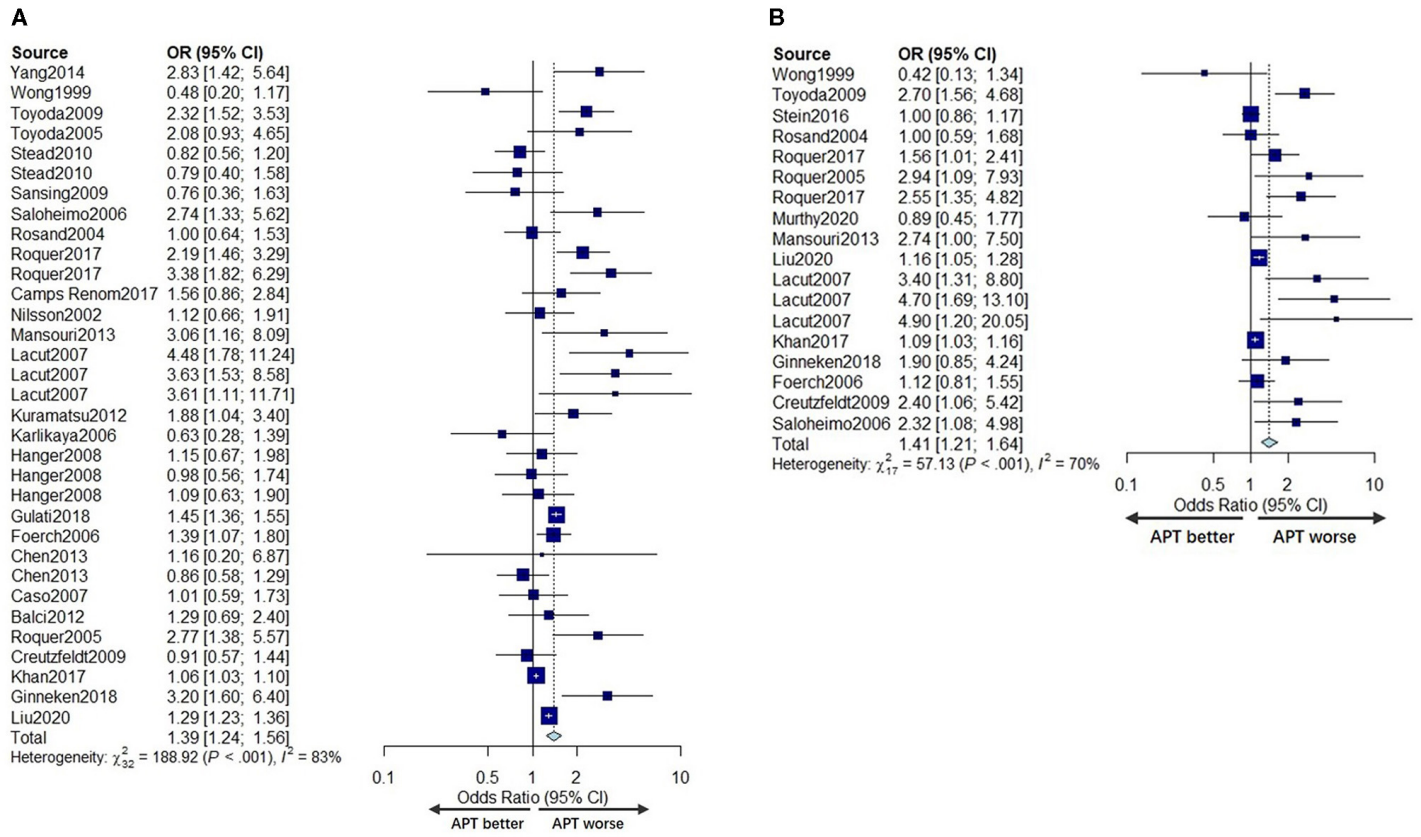
Forest plot comparing mortality for APT vs. no APT for univariate analyses **(A)** and multivariable adjusted analyses **(B)**.

The mortality of ICH patients was reported in 28 studies (5, 6, 8–13, 19, 21–35, 37–40) with 218,530 patients. Five studies (10, 28, 29, 33, 39) reported mortality at more than one time point. A total of 26 cohorts (210,842 patients) (6, 8–11, 13, 19, 21–35, 37–40) contributed data for the univariate mortality analysis, and 15 cohorts (203,969 patients) (5, 8, 9, 12, 13, 19, 23–26, 28, 30, 32, 37, 39) provided data for the multivariable adjusted mortality analysis. From both pooled univariate ORs and pooled multivariable adjusted ORs, we found that prior APT was significantly associated with higher mortality (OR 1.39, 95% CI 1.24–1.56; OR 1.41, 95% CI 1.21–1.64). However, substantial heterogeneity was detected for both univariate analyses and multivariable analyses (*I*^2^ = 83%, *P* < 0.001; *I*^2^ = 70%, *P* < 0.001) (Figures 3A,B).

To determine the source of heterogeneity, meta-regression analyses were conducted, and the results are presented in Tables 2, 3. The results revealed that the effect size was significantly correlated with different study centers, study types, and continents (*p* < 0.05) in univariate analyses and multivariable analyses.

**Table 2:** Meta-regression results of univariate analyses.

**Variables**		**Regression coefficient (SE)**	**95% CI**	***p-*value**
Published year		0.011	−0.0062–0.0369	0.1619
Population	Single center	0.0765	0.2292–0.5291	<0.0001^*^
	Multicenter	0.0901	0.0777–0.4308	0.0048^*^
Study type	Prospective	0.0932	0.0873–0.4526	0.0038^*^
	Retrospective	0.0758	0.2175–0.5145	<.0001^*^
Reputation		0.0443	−0.0353–0.1383	0.2451
Continent	America	0.1396	−0.3574–0.1897	0.5481
	Asia	0.1408	0.1247–0.6767	0.0044^*^
	Europe	0.0896	0.3175–0.6688	<0.0001^*^

* *p < 0.05*.

**Table 3:** Meta-regression results of multivariable adjusted analyses.

**Variables**		**Regression coefficient (SE)**	**95% CI**	***p-*value**
Published year		0.0138	−0.0456–0.0084	0.1777
Population	Single center	0.1204	0.4158–0.8878	<0.0001^*^
	Multicenter	0.0763	−0.0174–0.2817	0.0832
Study type	Prospective	0.1247	0.0118–0.5007	0.0399^*^
	Retrospective	0.1158	0.2352–0.6891	<0.0001^*^
Reputation		0.0534	−0.1620–0.0472	0.2821
Continent	America	0.2431	−0.3149–0.6381	0.5063
	Asia	0.2624	−0.1222–0.9065	0.1351
	Europe	0.1702	0.3138–0.9810	0.0001^*^

* *p < 0.05*.

##### Subgroup Analysis and Meta-Regression

To test the hypothesis that the assessment time could be an essential factor in mortality between APT and non-APT patients, we performed a series of subgroup analyses and meta-regression analyses based on the time of assessment.

**Figure 4:**
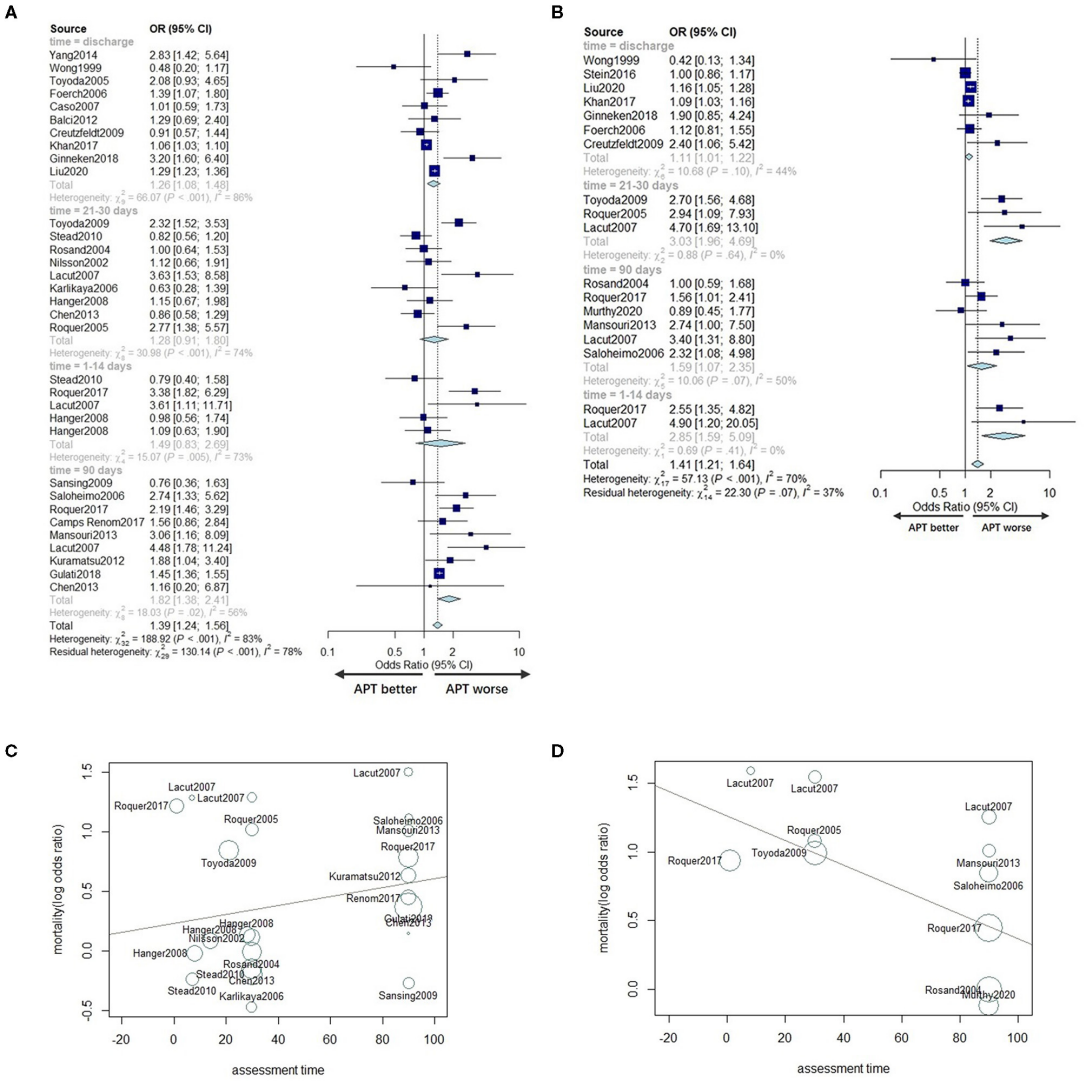
Subgroup analyses according to assessment time in univariate analyses **(A)** and adjusted analyses **(B)**. Meta-regression for the assessment time for univariate analyses **(C)** and multivariable adjusted analyses **(D)**.

In the subgroup analysis, we divided the included studies into four groups: early time (1–14 days), 30 days (21–30 days), 90 days, and discharge according to the evaluation time included. As shown in Figures 4A,B, the pooled unadjusted ORs for mortality were 1.49 (95% CI: 0.83–2.96), 1.28 (95% CI: 0.91; 1.80), 1.82 (95% CI: 1.38; 2.41), and 1.26 (95% CI: 1.08; 1.48) for each group, respectively. Similarly, the pooled adjusted OR for mortality of each group was 2.85 (95% CI: 1.59–5.09), 3.03 (95% CI: 1.96–4.69), 1.59 (95% CI: 1.07–2.35), and 1.11 (95% CI: 1.01–1.22), indicating that the relationship between prior APT use and mortality varied in different time periods.

To further explore the relationship between death events and assessment time, we conducted a meta-regression based on assessment time, excluding the time point at discharge for its variability. We found no significant association between the unadjusted OR for mortality and assessment time (*P* = 0.4216; Figure 4C). However, there was a significant trend regarding multivariable adjusted analyses, with the adjusted OR for mortality of APT patients decreasing by 0.0089 for each additional day of assessment time (95% CI: −0.0164 to −0.0015; *P* = 0.0192) (Figure 4D) compared to non-users.

#### Secondary Outcomes

##### Effects of Prior APT on the Outcome in ICH Patients

**Figure 5:**
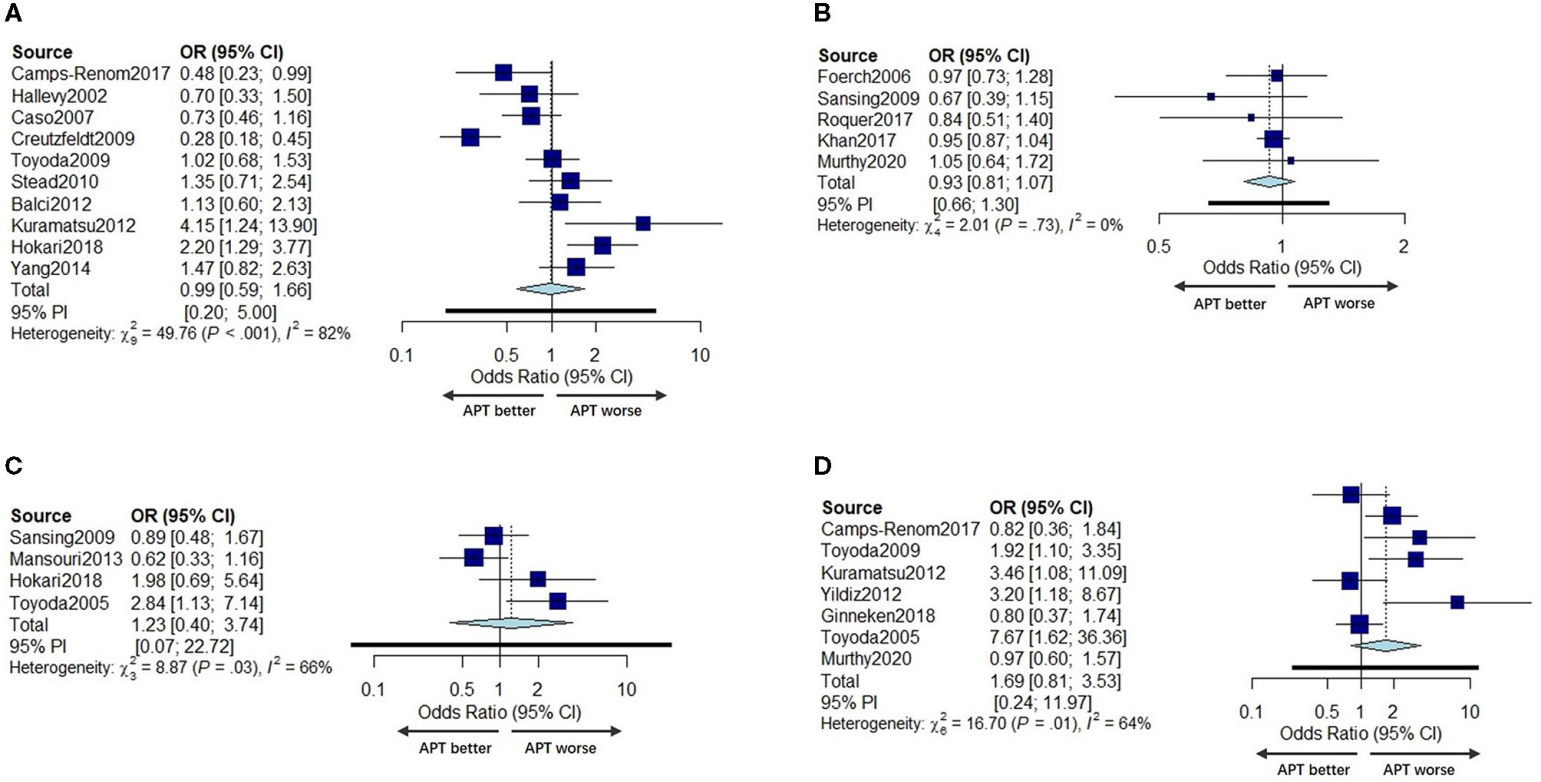
Forest plot of univariate OR **(A)** and multivariable adjusted OR **(B)** for poor function outcome in prior APT users compared to non-users. Forest plot comparing hematoma growth for APT vs. no APT in univariate analyses **(C)** and in multivariable adjusted analyses **(D)**.

Ten studies (11, 20, 27, 30, 32–35, 38, 41) with a total of 3,622 patients under univariate analyses and five studies (8, 12, 24, 31, 39) with a total of 86,201 patients under multivariable adjusted analyses reported the effects of prior APT on the poor functional outcome of ICH patients. Studies that did not report scale results were not included for poor prognosis analysis. No significant difference was found in the poor functional outcome between prior APT patients and no prior APT patients regarding either pooled unadjusted ORs or multivariable adjusted ORs (OR 0.99, 95% CI 0.59–1.66; OR 0.93, 95% CI 0.87–1.07) (Figures 5A,B). The between-study statistical heterogeneity was substantial for univariate analyses (*I*^2^ = 82%, *P* < 0.001).

##### Effects of Prior APT on Hematoma Growth in ICH Patients

Four studies with a total of 1,052 patients (6, 31, 37, 41) reported the effects of prior APT on HG with univariate ORs, and seven studies (6, 11–13, 32, 35, 36) reported this outcome with multivariable adjusted ORs (including 3,518 patients). The incidence of hematoma expansion was not significantly different between prior APT users and non-users in either univariate analyses or multivariable adjusted analyses (OR 1.23, 95% CI 0.40–3.74, OR 0.94, 95% CI 0.24–3.60) (Figures 5C,D). The statistical heterogeneity was also moderate (*I*^2^ = 66%, *P* = 0.03, *I*^2^ = 64%, *P* = 0.01).

##### Publication Bias and Sensitivity Analysis

**Figure 6:**
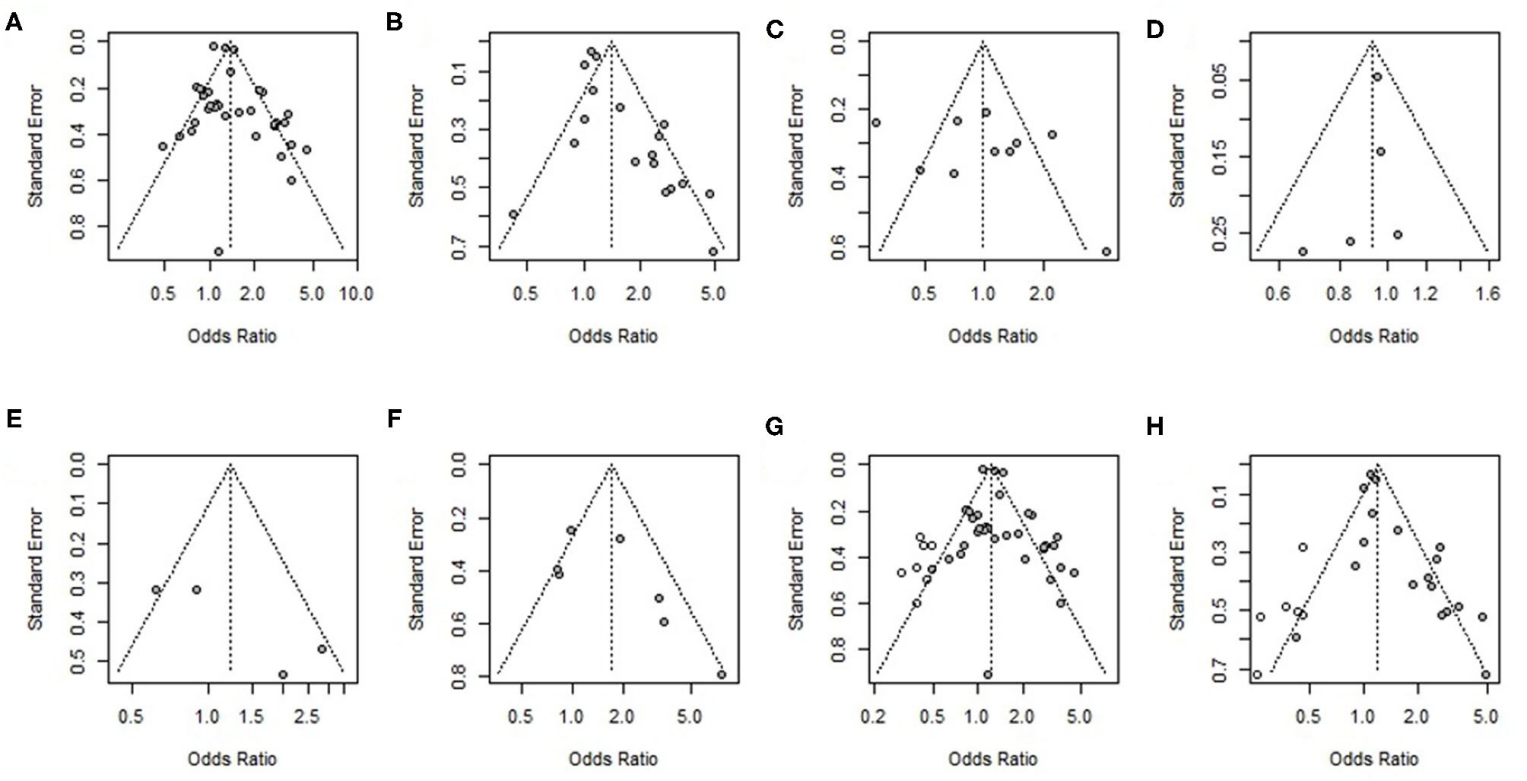
Funnel plot of **(A)** univariate odds ratios for mortality, **(B)** multivariable adjusted odds ratios for mortality, **(C)** univariate odds ratios for poor function outcome, **(D)** multivariable adjusted odds ratios for poor function outcome, **(E)** univariate odds ratios for hematoma growth (HG), **(F)** multivariable adjusted odds ratios for hematoma growth (HG), **(G)** results of the trim-and-fill analysis of univariate odds ratios for mortality, and **(H)** results of the trim-and-fill analysis of univariate odds ratios for mortality.

Funnel plots and Egger's-test were used to reveal possible publication bias. The results showed no overestimation of effect sizes except for the analysis of mortality in studies with univariate ORs and multivariable adjusted ORs (Egger's test: *P* = 0.4, *P* = 0.002) (Table 4). Next, the trim-and-fill method was applied to evaluate the impact of publication bias on our meta-analysis results. After seven studies and six studies were separately filled and no studies trimmed, the OR was not significantly changed (OR = 1.22, 95% CI 1.09–1.37, OR =1.21, 95% CI 1.02–1.42) (Figures 6G,H), suggesting that publication bias had little effect on the results. Funnel plots are shown in Figure 6.

**Table 4:** Egger's test of studies.

**Analysis**	**Egger's test (*p-*value)**	**Trim-and-fill estimate pooled-changes (95% CI)**
Univariate analyses for mortality	0.0398^*^	−0.17 (−0.15; −0.19)
Multivariable analyses for mortality	0.006^*^	−0.20 (−0.19; −0.22)
Univariate analyses for Poor function outcome	0.427	-
Multivariable analyses for Poor function outcome	0.449	-
Univariate analyses for hematoma growth	0.286	-
Multivariable analyses for hematoma growth	0.386	-

* *p < 0.05*.

## Discussion

### Summary of Main Results

The present meta-analysis was conducted to explore the effects of prior APT on mortality, functional outcome, and hematoma growth in patients with ICH. The meta-analysis demonstrated that prior APT users had a slightly increased mortality. However, prior APT had no effects on the poor functional outcome or hematoma growth in patients with ICH.

### Primary Outcome

Clarifying the relationship between prior APT and ICH mortality is important because a large portion of the general population regularly takes these drugs, and their usage is likely to increase as the population ages (42, 43). Furthermore, restoration of normal platelet function could be a therapeutic target if prior APT worsens the outcome of ICH patients. This present meta-analysis showed that prior APT led to increased mortality, as reported by a previous study (4). The determination of mortality has high between-group reliability and is less susceptible to the determination bias associated with the study design. Moreover, the pooled results in this meta-analysis were consistent with both unadjusted ORs and adjusted ORs. Although it is difficult to completely eliminate the influence of all confounding factors, the above factors indicate the high reliability of our results.

The included studies in previous reviews (4) were published from 1998 to 2010, and the majority of them were studies published around 2005, which is a long time ago. Fifty-three percent of the studies (5, 8–13, 34–41) included in our present meta-analysis were published after 2010, which reported conflicting results. Therefore, an updated meta-analysis is needed. Additionally, we collected data with more assessment time points, such as early access at 30 days before, and conducted a meta-regression analysis based on assessment time to further explore whether the relationship between prior APT use and mortality changed over time of assessment. Based on the above analysis, we also analyzed the relationship between APT and hematoma dilatation.

In the subgroup analysis, the results indicated that early-time death was more frequent in patients on prior APT in multivariable adjusted analyses, the same as that at 90 days. However, at 30 days, the relationship became insignificant. These results were similar to those reported by Roquer's prospective study (39). Roquer believed that the higher mortality of prior APT patients was related to the strong influence of APT pretreatment on early mortality, and 90-day mortality seems to be a subrogation of early-time mortality. Therefore, to verify the above hypothesis, we performed a further meta-regression analysis to explore the linear relationship between assessment time and the effect of prior APT use on mortality and found consistent results. We found that the effect of prior APT on mortality use decreased over time in multivariable adjusted analyses. Since the platelet life was 7–10 days, with an ~10% rate of daily updates (44), prior APT patients who present with higher early-time mortality are believed to have insufficient platelet activity early in life (6, 26, 32). However, in univariate analyses, death was significantly more frequent in patients on prior APT only in the 90-day group, and no significant association was found. This difference might be explained by the fact that patients pretreated with APT were older and had poor previous functional status and more vascular risk factors than the non-pretreated group (5, 9–13, 24–36, 38, 39). In addition, long-term discontinuation of APT may worsen cardiovascular and cerebrovascular conditions and lead to death. The selection of APT reuse time for ICH patients should be cautious because prematurely resuming antiplatelet therapy may potentially increase ICH recurrence risk, whereas unnecessarily delaying the restart of antiplatelet therapy may significantly increase the patient's risk of thromboembolism, and many relative clinical studies are still needed (45, 46). Meanwhile, given the increase in risk, whether platelet function reversal strategies can ameliorate the mortality associated with pre-ICH APT at an early time would require relatively large trials to demonstrate. More medical attention should be given to ICH patients with prior APT use.

We found statistical evidence of heterogeneity in both univariate analyses and multivariable adjusted analyses, and our univariate meta-regression analysis showed that the difference in study range, study types and continents could be the primary reasons for heterogeneity—different patients exhibit differential drug sensitivities, and different regions have different drug preferences (3). Drug sensitivity, different types of APT (9, 47), dual or triple APT use (8), and the duration or dosage of APT could influence the outcome of ICH (8, 39). In addition, the heterogeneity of the adjusted OR could also be ascribed to the different adjusted factors in the multivariate analysis of each study. Finally, the inherent biases and differences in the designs of the observational studies lead to an increased risk of heterogeneity.

### Secondary Outcomes

In addition to mortality, functional outcome is also a hot topic in research. We found that prior APT did not play an unfavorable role in the prognosis of ICH, and the result was consistent with that of Thompson et al. (4).

Moreover, we found that prior APT was not associated with early hematoma growth (HG). All this suggests that hematoma growth may not be a possible mechanism by which prior APT causes higher mortality in ICH patients, which contradicts the results reported by Camps-Renom (11). One possible explanation regarding the discrepancy among studies is that we included more studies with larger sample sizes and separated unadjusted ORs and adjusted ORs for statistical analysis. In addition, another possible explanation may be that platelet activity is not measured directly but inferred from the medical history (11, 13). There may be a threshold effect where the reduction in platelet activity must be substantial enough to influence the outcome (8). Therefore, in the future, further research can conduct more in-depth exploration by directly detecting the platelet activity of patients.

### Sensitivity Analysis and Publication Bias

Sensitivity analysis indicated that the results of this study were reliable. However, it should be admitted that the quality of the included studies was indeed at a medium level, which is related to the fact that all studies were non-RCT observational studies. Fifty-seven percent of the included studies were retrospective studies, and some studies were secondary studies. The quality of evidence was downgraded mainly by the retrospective design of the studies. Statistical analysis of patients' prior APT use was accompanied by an inevitable recall bias. Furthermore, due to too many related confounding factors, it is difficult to obtain comprehensive statistics in the studies.

Notably, publication bias indeed exists in the analysis of mortality in studies with adjusted ORs. The results of the trim-and-fill analysis showed that there were no significant changes in the estimate of the combined effect size. Publication bias had little effect on the results, and the results were robust.

Several studies compared the effects of different types of antiplatelet agents on the outcomes in ICH patients. However, the results were not appropriate to be pooled due to the significant heterogeneity. Toyoda et al. (32) compared the effects of aspirin, other single APT use, and dual APT use and found that aspirin use was associated with more 30-day mortality and hematoma enlargement; Liu et al. (9) compared the effects of cyclooxygenase inhibitor (COX-I), adenosine diphosphate receptor inhibitor (ADP-I), and phosphodiesterase inhibitor (PDE-I) and found that ADP-I and COX-1 are the most likely contributors to the poor outcomes in spontaneous ICH patients. Khan et al. (8) suggested that the previous use of CAPT, but not SAPT, was associated with a higher risk of in-hospital mortality among ICH patients. Further studies are needed to explore the effects of different choice, usage, and dosage of antiplatelet agents on the outcomes in ICH patients.

### Limitations

The current study has several limitations. First, all the included studies were non-RCT observational cohort studies. Second, data regarding the choice, usage, and dosage of antiplatelet agents were not appropriate to process correlated analysis due to the significant heterogeneity. Large sample RCTs are needed to evaluate these.

## Conclusion

### Implications for Practice

The present study represents that prior APT was not associated with hematoma expansion or functional outcomes, but there was modestly increased mortality in prior APT patients. Safety concerns should be considered when chronic antiplatelet treatment is planned. Additionally, the finding that higher mortality of prior APT patients was related to the strong influence of prior APT use on early mortality suggested that early-time stage in ICH patients with prior APT is crucial, which needs close monitoring and management.

### Implications for Research

Whether it is possible to reduce prior APT mortality in ICH patients by restoring early platelet function requires relatively large trials to demonstrate. In addition, our conclusion negates the correlation between prior APT and hematoma expansion; therefore, whether prior APT use could be an independent predictor of early hematoma growth (HG) still needs further exploration. Further research can conduct more in-depth exploration by directly detecting the platelet activity of patients.

## Data Availability Statement

The original contributions presented in the study are included in the article/supplementary material, further inquiries can be directed to the corresponding authors.

## Author Contributions

All authors listed have made a substantial, direct and intellectual contribution to the work, and approved it for publication.

## Conflict of Interest

The authors declare that the research was conducted in the absence of any commercial or financial relationships that could be construed as a potential conflict of interest.

## Publisher's Note

All claims expressed in this article are solely those of the authors and do not necessarily represent those of their affiliated organizations, or those of the publisher, the editors and the reviewers. Any product that may be evaluated in this article, or claim that may be made by its manufacturer, is not guaranteed or endorsed by the publisher.
